# Physico-Chemical Parameters, Phenolic Profile, In Vitro Antioxidant Activity and Volatile Compounds of Ladastacho (*Lavandula stoechas*) from the Region of Saidona

**DOI:** 10.3390/antiox8040080

**Published:** 2019-03-28

**Authors:** Ioannis K. Karabagias, Vassilios K. Karabagias, Kyriakos A. Riganakos

**Affiliations:** Laboratory of Food Chemistry, Department of Chemistry, University of Ioannina, 45110 Ioannina, Greece; vkarambagias@gmail.com (V.K.K.); kriganak@uoi.gr (K.A.R.)

**Keywords:** Ladastacho, characterisation, properties, HPLC/ESI-MS, HS-SPME/GC-MS, beneficial use

## Abstract

The aim of the present study was to characterize *Lavandula stoechas* (Ladastacho) from the region of Saidona by means of physico-chemical parameters, phenolic profile, in vitro antioxidant activity and volatile compounds. Physico-chemical parameters (pH, acidity, salinity, total dissolved solids, electrical conductivity and liquid resistivity) were determined using conventional methods. The phenolic profile was determined using high-performance liquid chromatography electrospray ionization mass spectrometry (HPLC/ESI-MS), whereas a quantitative determination was also accomplished using the total phenolics assay. In vitro antioxidant activity was determined using the 2,2-diphenyl-1-picryl-hydrazyl assay. Finally, volatile compounds were determined using headspace solid phase microextraction coupled to gas chromatography mass spectrometry (HS-SPME/GC-MS). The results showed that *Lavandula stoechas* aqueous extract had a slightly acidic pH, low salinity content and considerable electrochemical properties (electrical conductivity and liquid resistivity along with electric potential). In addition, aqueous fractions showed a significantly (*p* < 0.05) higher phenolic content and in vitro antioxidant activity, whereas phenolic compounds, such as caffeic acid, quercetin-*O*-glucoside, lutelin-*O*-glucuronide and rosmarinic acid, were identified. Finally, numerous volatile compounds were found to dominate the volatile pattern of this flowering plant, producing a strong, penetrating, cool and menthol-like odour.

## 1. Introduction

The systematic cultivation and exploitation of the health benefits and beneficial applications of historical flowering plants/herbs is of great value for the modern world as new challenges arise day-by-day. The most important challenge, however, is the maintenance of the welfare and healthcare of humans in lieu of the use of synthetic drugs that may cause adverse effects.

Indeed, since ancient times, there has been all over the world a systematic use of herbs for the treatment of several health disorders. For example, in Greece, such herbs as oregano, marjoram, dill, fennel, mint, rosemary, mountain tea, sage, chamomile, thyme, parsley and basil have been used not only to flavour the cuisine or traditional dishes but also for medicinal purposes. Another important flowering plant that has a long history is Lavandula or, more commonly, lavender.

Approximately 47 species of the Lavandula genus are known, belonging to the mint family Lamiaceae. These flowering plants have a long history and have been found in numerous regions of the world, including Europe, the Mediterranean zone, Africa, Asia and India [1]. The temperate climates favor the cultivation of *Lavandula* spp. as ornamental plants or culinary herbs, and also at a commercial level for the extraction of essential oils [2]. Lavandula is grown in well-drained soils and in light places with good air circulation. It cannot grow in the shade [3,4]. The suitable soil pH may vary from acid to neutral and basic, whereas lavender can favorably grow in very alkaline soils. The optimum pH for the growth of Lavandula is between 6 and 8 [5]. In most cases, lavender is harvested by hand, depending on the usage [5].

In the Greek and Mediterranean zones, the species that grow originally are *Lavandula stoechas* [6]. Furthermore, the species of *Lavandula stoechas*, *Lavandula pedunculata* and *Lavandula dentata* were reported in Roman times [7]. The most commonly cultivated plant is the English lavender *Lavandula angustifolia* (formerly named *L. officinalis*). Some other commonly grown ornamental species are *Lavandula stoechas*, *Lavandula dentata* and *Lavandula multifida* (Egyptian lavender).

The lavender flower has been reported to have plenty of uses in practices of herbalism. The German scientific committee enhanced its use in alternative medicine, including its use for restlessness or insomnia, Roehmheld’s syndrome, intestinal discomfort and cardiovascular diseases, among others [8]. The National Institutes of Health (NIH) in Maryland, U.S., stated that lavender is considered ‘’likely’’ safe in food amounts and ‘’possibly’’ safe in medicinal amounts. However, the NIH did not recommend the use of lavender in pregnant or breast-feeding women because of a lack of knowledge of its effects. Lavender oil should not be used in young boys due to the possible hormonal effects leading to gynecomastia [9,10]. In addition, the NIH stated that lavender may cause skin irritation and could be poisonous if consumed orally [11]. In 2005, a review on lavender essential oil reported that lavender is traditionally regarded as a ’safe’ oil, whereas skin problems (contact dermatitis) may occur at only a very low frequency [12]. In a study dealing with the relationship between various fragrances and photosensitivity carried out in 2007, it was reported that, even though lavender is known to favour cutaneous photo-toxic reactions, it did not induce photohaemolysis [13].

Saidona is a beautiful village in the Messiniaki Mani. Organic olive oil, table olives, sage, mountain tea, *Lavaldula stoechas*, and other flowering plants flourish in considerable amounts in Saidona. There is a long historical tradition of the local citizens using *Lavandula stoechas*, commonly referred as ‘’ladastacho’’, for the treatment of hair loss by preparing a gently boiled aqueous solution of its flowers.

Based on the above, the aim of the present work was to investigate the aqueous and methanolic extracts of *Lavandula stoechas* in terms of phenolic profile and in vitro antioxidant activity along with volatile compounds; some physico-chemical parameters were also considered. To the best of our knowledge, this is the first report in the literature that reports a combination of different parameter analyses for the characterisation of such a flowering plant grown in the Greek zone.

## 2. Materials and Methods

### 2.1. Collection of Lavandula stoechas and Preparation of Samples

Two kilograms (kg) of *Lavandula stoechas* was collected from the region of Saidona, Mani, (Messinia, Greece) (36°52′55″N 22°17′01″E) during June of 2017. *Lavandula stoechas* is shelf-grown in this region without the use of any cultivation system/procedure. The flowers were left alone to dry in a dark place and were then gently removed from the body of the plant. Finally, these were stored in aluminum foil at 4 ± 1 °C until further analysis.

### 2.2. Reagents and Solutions

Gallic acid (3,4,5-trihydrobenzoic acid) anhydrous for synthesis was purchased from Merck (Darmstadt, Germany). Methanol for analysis, Folin–Ciocalteu phenol reagent, sodium chloride (NaCl), sodium hydroxide (NaOH) and sodium carbonate (Na_2_CO_3_) were purchased from Merck. Finally, 2,2-Diphenyl-1-picrylhydrazyl (DPPH) was purchased from Sigma-Aldrich (Darmstadt, Germany).

### 2.3. Physicochemical Parameter Analysis

#### 2.3.1. Determination of Acidity

For the determination of acidity, 10 g of *Lavandula stoechas* was transferred to a conical flask and 100 mL of distilled water was added. The obtained solution was titrated with a standard solution of sodium hydroxide (0.1 N) after the addition of a few drops of phenolphthalein, serving as an indicator of equivalent point. The results reported are the average values ± standard deviation values of three replicates (*n* = 3) and are expressed as g NaOH per 100 g of dried *Lavandula stoechas* [14].

#### 2.3.2. Determination of pH

pH values of *Lavandula stoechas* were measured using a Delta OHM, model HD 3456.2, pH-meter (Delta OHM, Padova, Italy) (precision of ± 0.002). Samples (10 g) were diluted with 100 mL of distilled water and the homogenate was used for the pH determination. Reported results are the average values ± standard deviation values of three replicates (*n* = 3). All measurements were carried out at 15 ± 1 °C after immersion of the electrode (that was firstly cleaned with distilled water) in the herb aqueous solution, until constant values were reached.

#### 2.3.3. Determination of Electrical Conductivity, Salinity and Total Dissolved Solids

Electrical conductivity, liquid resistivity, salinity and total dissolved solids of 10% (*w v*^−1^) aqueous herb solutions in distilled water were measured using a Delta OHM, model HD 3456.2, conductimeter (Delta OHM, Padova, Italy) with four-ring and two-ring conductivity/temperature probes. Temperature was measured by four-wire Pt100 and two-wire Pt1000 sensors by immersion. The probe was calibrated automatically using a 1413 μS cm^−1^ conductivity standard solution (Hannah Instruments, Inc., Woonsocket, RI, USA). Results are expressed as mS cm^−1^, Ohm (Ω), g L^−1^ and mg L^−1^, respectively. The results reported are the average ± standard deviation values of three replicates (*n* = 3).

#### 2.3.4. Extraction of Phenolic Compounds

Extraction of phenolic compounds was carried out in two independent extraction systems: methanol and water. In particular, 0.34 g of dried and gently blended *Lavandula stoechas* was placed in plastic volumetric flasks containing 30 mL of methanol or water (1.13% (*w v*^−1^), (11,333.33 mg L^−1^)). The vials were then centrifuged for 2 h at 4000 rpm. The supernatant was filtered using a filtrate paper and the extracts were collected in plastic vials, having been wrapped with parafilm and aluminum foil. Finally, these were kept at −18 °C until analysis and used for the determination of phenolic compounds and in vitro antioxidant activity.

#### 2.3.5. Analysis of *Lavandula stoechas* Phenolic Compounds Using High-Performance Liquid Chromatography Electro Spray Ionization Mass Spectrometry (HPLC/ESI-MS)

An Agilent, model 1100 series, HPLC system (Agilent, Santa Clara, California, USA) was used for the chromatographic analysis. The respected wavelength was that of 280 ± 2 nm. Water and acetonitrile (Merck, Darmstadt, Germany) were used as the mobile phase at a flow rate of 1 mL min^−1^. The gradient elution program followed the sequence: 10% of acetonitrile then increasing to 30% for 20 min, further increasing to 40% at 30 min, to 50% at 35 min, and finally to 50% at 40 min. To avoid any memory effects, the column was eluted isocratically for 10 min before the next injection. A clear separation of the phenolic compounds was achieved using the Eclipse XDB C8 column (250 mm × 4.6 mm × 5 μm, Agilent, Santa Clara, USA) at 25 °C.

The mass spectrometer was the LC/MSD trap SL (Agilent). The MS conditions were as follows: Injection volume: 3.5 μL; Source conditions: Drying gas (nitrogen) 8 L/min at 330° C; Nebulizer pressure: 50 psi; Mass range: 100–1000; Scan mode: negative (−). Identification of phenolic compounds was achieved by comparing the mass to charge values [Μ−Η]- of individual peaks shown at total ion chromatograms with those identified previously in the literature. Analysis of *Lavandula stoechas* samples was run in duplicate (*n* = 2).

#### 2.3.6. Determination of in Vitro Antioxidant Activity

Preparation of DPPH free radical standard solution

A standard solution of [DPPH•] equal to ca. 0.08 mM (mmol L^−1^) was prepared by dissolving 0.031 g of the free radical in 100 mL of methanol.

#### 2.3.7. Preparation of the DPPH Free Radical Calibration Curve

A calibration curve of concentration versus absorbance of [DPPH•] was prepared as follows: The aforementioned standard solution of DPPH was diluted with the addition of methanol. The resulting solutions (range of 0–31 mg L^−1^) were vortexed, left in the dark (until measurements were made), and the absorbance was measured in a UV/VIS Spectrometer (Lambda 25, PerkinElmer, Waltham, USA) at λmax of 517 nm. The calibration curve of absorbance (*y*) versus concentration (*x*) of [DPPH•] was expressed by the following equation:

*y* = 0.0243*x* − 0.0001; *R*^2^ = 0.998 (1)

#### 2.3.8. Determination of in Vitro Antioxidant Activity of *Lavandula stoechas* Aqueous and Methanolic Extracts

The antioxidant activity of *Lavandula stoechas* aqueous and methanolic extracts was estimated in vitro using the [DPPH•] assay. For the determination of antioxidant activity, a volume of 3.0 mL of [DPPH•] solution (0.08 mM) was placed in the cuvette (final volume of 3 mL) and the absorbance of the [DPPH•] radical was measured at *t* = 0 (*A*_0_ = 0.7565). Subsequently, 0.20 mL of *Lavandula stoechas* aqueous and methanolic extracts was placed in the respective cuvettes plus 2.8 mL of the [DPPH•] solution. The absorbance was measured every 15 min (regular time periods) until the value reached a plateau (steady state, *A_t_*). The reaction between the free radical and *Lavandula stoechas* water soluble and methanolic antioxidants was accomplished at 30 min. The absorbance of the reaction mixture was measured at 517 nm.

The [DPPH•] antioxidant activity (%AA) with respect to aqueous and methanolic extract of *Lavandula stoechas* was calculated using the following equation:

%*AA* = ((*A*_0 −_*A_t_*)/*A*_0_) × 100 (2)

where *A*_0_ is the initial absorbance of the [DPPH•] free radical standard solution and *A_t_* is the absorbance of the remaining [DPPH•] free radical after reaction with *Lavandula stoechas* antioxidants, at steady state (plateau). For the estimation of *Lavandula stoechas* effective concentration EC_50_, termed as the concentration of antioxidants that could cause a decrease in the DPPH free radical by 50%, further dilutions from the initial extracts (11,333.33 mg L^−1^) were prepared for methanolic and aqueous extract from the initial mother solution in the range 500–11,333.33 mg L^−1^ and the value of EC_50_ was obtained from graphs of [DPPH•] remaining versus concentration of extracts. For this antioxidant test, methanol was used as the blank. Prior to absorbance measurements, the extracts were filtered using Whatman filters (Whatman plc, Buckinghamshire, UK) with a pore size of 0.45 μm. Each sample was run in triplicate (*n* = 3).

#### 2.3.9. Determination of Total Phenolic Content

The total phenolic content of *Lavandula stoechas* was determined in its aqueous and methanolic extracts using the Folin-Ciocalteu assay according to Singleton et al. [15]. In particular, in a 5 mL volumetric flask, 0.2 mL of herb extract, followed by 2.5 mL of distilled water and 0.25 mL of Folin-Ciocalteu reagent, were added. After 3 min, 0.5 mL of saturated Na_2_CO_3_ (30%, *w v*^−1^) was also added and the final volume of 5 mL was reached using distilled water. The flask was then stored in a dark place and the absorbance was measured after 2 h at 760 nm in a UV/VIS Spectrophotometer (SHIMADZU, UV-1280, Kyoto, Japan). Before any absorbance measurements, the solutions were filtered using Whatman filters (Whatman plc, Buckinghamshire, UK) with a pore size of 0.45 μm. For the quantification analysis, a calibration curve of standard gallic acid was constructed in wide range of concentrations: 0–6240 mg L^−1^. The equation obtained was of the following form:

*y* = 0.0004*x* + 0.1479, *R*^2^ = 0.9895 (3)

Total phenolic content was expressed as mg of gallic acid equivalents per mL (or g L^−1^) of *Lavandula stoechas* extract. Each sample was analysed in triplicate (*n* = 3).

## 3. Headspace Solid Phase Microextraction Coupled to Gas Chromatography/Mass Spectrometry (HS-SPME/GC-MS)

### 3.1. Headspace Extraction of Volatile Compounds

The extraction of volatile compounds of *Lavandula stoechas* was carried out using a divinyl benzene/carboxen/polydimethylsiloxane (DVB/CAR/PDMS) fiber of 50/30 μm purchased from Supelco (Bellefonte, PA, USA). Before analysis of samples, the fiber was conditioned according to the manufacturer’s recommendations and was cleaned daily using the method of ‘’clean’’ program to avoid any source of contamination. In particular, the injector and MS-transfer line were maintained at 260 °C and 270 °C, respectively, whereas during the ‘’cleaning’’ of the fiber, the oven temperature was held at 80 °C for 0 min, and then was increased to 270 °C at 10 °C per min (2 min hold). A split ratio of 10:1 was used.

For the sample analysis, the following conditions were followed: 15 min equilibration time, 30 min sampling time, and 45 °C water bath temperature. Approximately, 0.05 g of *Lavandula stoechas* was then placed in a 15 mL vial equipped with a polytetrafluoroethylene PTFE/silicone septa screw-cap, along with a magnetic stirrer. The vials were introduced into a water bath of 45 °C under continuous stirring (600 rpm) during the headspace extraction [16].

### 3.2. Gas Chromatography-Mass Spectrometry Unit and Analysis Conditions

The GC unit used in the study for the gas chromatography/mass spectrometry analysis of *Lavandula stoechas* samples was an Agilent 7890A model coupled to a MS detector (Agilent 5975, Agilent, Santa Clara, USA). The DB-5MS capillary column (Agilent, Santa Clara, USA) was used in the analysis with the following characteristics: cross-linked 5% PH ME siloxane, of 60 m × 320 μm i.d., × 1 μm film thickness. Helium of excellent purity (99.999%) was the carrier gas at a flow rate of 1.5 mL min^−1^. The injector and MS-transfer line were maintained at 250 °C and 270 °C, respectively, whereas, during the analysis, the oven temperature was maintained at 40 °C for 3 min, and then was increased to 260 °C at a rate of 8 °C min^−1^ (6 min hold). The electron impact mass spectra were recorded at a mass range of 50–550, and the ionization energy was 70 eV, whereas a split ratio of 1:2 was used to introduce the appropriate amount of sample onto the column [16].

### 3.3. Identification of *Lavandula stoechas* Volatile Compounds

The identification of volatile compounds was achieved using the Wiley 7, NIST 2005 mass spectral library (John Wiley & Sons, New York, USA). The Kovats indices were determined by the use of a mixture of n-alkanes (C8–C20) supplied by Supelco (Bellefonte, PA, USA). Prior to gas chromatography-mass spectrometry (GC/MS) analysis, the mixture was dissolved in n-hexane and the retention time of standards was determined according to the aforementioned temperature-programmed run. Only volatile compounds having ≥80% similarity with the Wiley library were tentatively identified using the GC-MS spectra. The method of identification was based on the combination of MS data found in the Wiley 7 NIST 2005 mass spectral library and data of Kovats index values that were determined for each volatile compound and then compared with those included in the Wiley MS library [16]. Data were expressed as a percentage (contribution of the area of each volatile to the total peak area multiplied by 100). Volatile compounds identified only in replicated samples were used in the study.

### 3.4. Graph Preparation and Statistical Analysis

Graphs and average ± standard deviation values of physico-chemical parameters, in vitro antioxidant activity, total phenolic content and volatile compounds were prepared using the SPPS statistics software (v.20, 2013, IBM, New York, USA) and Microsoft Office Excel spread sheets for Windows 2007 (Microsoft, Redmond, USA). A t-test and Pearson’s bivariate statistics were carried out using the SPPS statistics software. The level of significance was considered to be that of *p* ≤ 0.05.

## 4. Results and Discussion

### 4.1. Physico-Chemical Parameter Analysis

The pH of *Lavandula stoechas*, showing the active acidity of the sample, was slightly acidic in the range of 5.74 ± 0.03. The electric potential (E0), which in our case could show how the electric potential energy of any charged bio-molecule(s) at any location in the solution is divided by the charge of that/those molecule(s), was 73.4 ± 0.2 (mV). In addition, low salinity content was observed (0.29 ± 0.00 g L^−1^) and the total dissolved solids content was in the range of 280 ± 2 mg L^−1^. The electrical conductivity, a measure that defines the charged molecules in an aqueous medium, was 579 ± 1 μS cm^−1^ and the liquid resistivity, a measure that defines how strongly a material opposes the flow of electric current, was 1780 ± 20 Ω (Ohm). Finally, free acidity was in the range of 33 ± 3 g NaOH per 100 g of dried *Lavandula stoechas*. To our knowledge, the data are scarce on the physicochemical parameters of *Lavandula stoechas* aqueous extracts. Therefore, comparisons with literature data cannot be provided. In the work of Murthy et al. [14], it was reported that *Lavandula bipinnata* seed oil showed a much lower acidity: ca. 5.76 mg NaOH per g.

### 4.2. Phenolic Profile: Qualitative and Quantitative Determinations

#### 4.2.1. Qualitative Analysis

**Figure 1:**
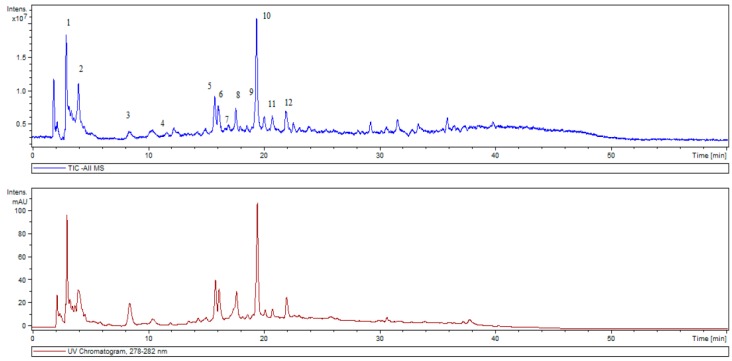
Total ion and UV chromatograms of *Lavadula stoechas* aqueous extract. Phytochemicals are numbered according to the retention time shown in Table 1.

The HPLC-ESI/MS analysis enabled the determination of 12 phytochemicals as shown in Table 1. Figure 1 shows a typical total ion and UV chromatogram of the aqueous *Lavandula stoechas* extract pointing out with numbers the identified phytochemicals according to retention time.

Caffeic acid has been previously reported to be the main natural phenol in argan oil [17]. It has also been found in the freshwater fern *Salvinia molesta* [18], in the mushroom *Phellinus linteus* [19], in the bark of *Eucalyptus globulus* [20] and in barley grain [21]. Its antioxidant potential has been reported in vitro and also in vivo [22]. In addition, caffeic acid has been reported to possess an inhibitory effect on cancer cell proliferation in human HT-1080 fibro-sarcoma cells [23]. Despite its antioxidant potential, there are some studies that report adverse results regarding the carcinogenicity or anti-carcinogenicity of caffeic acid [24].

Rosmarinic acid is mainly found in culinary herbs, such as *Ocimum basilicum* (basil), *Ocimum tenuiflorum* (holy basil), *Melissa officinalis* (lemon balm), *Rosmarinus officinalis* (rosemary), *Origanum majorana* (marjoram) and *Salvia officinalis* (sage), thyme and peppermint or in plants with medicinal properties, such as common self-heal (*Prunella vulgaris*) or in species of the genus Stachys [25]. It is also found in other Lamiales, such as *Heliotropium foertherianum*, a plant in the family Boraginaceae. Rosmarinic acid accumulation is favored in hornworts in the fern family Blechnaceae and in species of several orders of mono- and dicotyledonous angiosperms [26], which may be found also as a derivative in Anthocerotophyta (*Anthoceros agrestis*) in the form of rosmarinic acid 3′-*O*-β-d-glucoside [27].

Regarding some potential health effects, rosmarinic acid is a potential anxiolytic as it acts as a gamma-aminobutyric acid (GABA) transaminase inhibitor, more specifically on 4-aminobutyrate transaminase [28]. Rosmarinic acid also inhibits the expression of indoleamine 2,3-dioxygenase via its cyclooxygenase-inhibiting properties [29], whereas it was found to be effective in a mouse model of Japanese encephalitis [30].

Salvianolic acid B (Sal B) is a phenolic acid derived from the dried root and rhizome of *Salvia miltiorrhiza* (Labiatae), which has been used traditionally in most Asian countries for the clinical therapy of various vascular diseases for hundreds of years. Salvia has been also used for various diseases related to blood stasis syndrome in China for thousands of years, and now it is widely used for cardiovascular diseases (CVDs) [31]. In addition, Sal B protects diverse kinds of cells from damage caused by a variety of toxic stimuli [32].

Furthermore, flavonoids may also have numerous beneficial effects in human health. For instance, apigenin and the substituted derivatives (i.e., -apigenin-7-*O*-glucuronide) isolated from the *Marrubium deserti* de Noé plant have been reported to possess antioxidant and antigenotoxic properties [33]. The same holds for Luteolin 7-glucoside, often called cynaroside, which is a flavone. It has been identified in *Campanula persicifolia* and *Campanulla rotundifolia* [34], *Teucrium gnaphalodes* [35], *Ferula varia* and *Ferula foetida* [36], the bamboo *Phyllostachys nigra* [37], dandelion coffee, *Cynara scolymus* (artichoke) [38] and *Phoenix hanceana* var. formosana [39]. What is remarkable is that antioxidant and neural cell protective effects have also been reported for the latter ethanolic extract, in which the cynaroside was identified [39].

#### 4.2.2. Quantitative Analysis: Total Phenolic Content

The total phenolic content of the aqueous extract of *Lavandula stoechas* recorded a much higher value (ca. 4289 mg L^−1^) compared to the methanolic one (ca. 217 mg L^−1^), indicating that the aqueous medium (higher polarity) favoured the effective release/isolation of phytochemicals, especially phenolic acids and flavonoids, among other reducing agents (i.e., ascorbic acid). In a recent work [40], the preparation of *Lavandula angustifolia* beverages with either the method of infusion or decoction resulted in a beverage with a higher phenolic content when water, among other solvents, was used for the extraction of phytochemicals, in agreement with the present results.

The alcoholic and water extracts (1:1, *v*:*v*) of Romanian *Lavandula angustifolia* resulted in a significantly lower total phenolic content expressed as gallic acid (GA) equivalents (50.6 ± 3.2 mg GA g^−1^) compared to the results of the present study [41].

#### 4.2.3. In Vitro Antioxidant Activity

**Figure 2:**
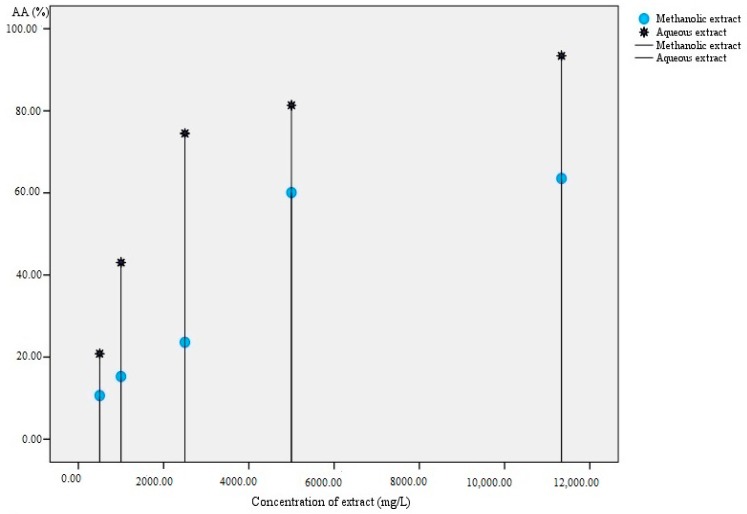
Antioxidant activity (AA%) of *Lavavdula stoechas* with respect to extract concentration (mg L^−1^).

Both methanolic and aqueous extracts of *Lavandula stoechas* showed in vitro antioxidant activity during the DPPH assay. However, the aqueous extract (of higher polarity) recorded a significantly (*t* = −4.196, *df* = 4, *p* = 0.014) higher antioxidant activity. In addition, higher antioxidant activity was recorded for the higher concentration of the extracts (Figure 2). The EC_50_ values were 7.05 mg mL^−1^ and 1.78 mg mL^−1^ for the methanolic and aqueous extracts, respectively.

What is also of great importance is that there was a perfect correlation between the in vitro antioxidant activity and the total phenolic content of the methanolic and aqueous extracts using Pearson’s bivariate statistics (*r* = 1, *p* = 0.01). The present results are in agreement with those of Hu et al. [37], who reported that solvent-extracted bamboo leaf extract (BLE) containing chlorogenic acid, caffeic acid and luteolin 7-glucoside showed in vitro antioxidant activity using the DPPH assay (among others). The authors also reported that BLE exhibited a concentration-dependent scavenging activity of the [DPPH•], in agreement with the present results.

*Lavandula angustifolia* extracts from the region of Southeast Romania showed a considerable in vitro antioxidant activity against the DPPH free radical, as reported by Spiridon et al. [41].

In a more recent work, *Lavandula angustifolia* (from the region of Polykarpi Pozar in Greece) aqueous extracts prepared with infusion or decoction also showed a high in vitro antioxidant activity against the DPPH free radical (>90%), in agreement with the present results [40].

#### 4.2.4. Volatile Compounds of *Lavandula stoechas*

**Figure 3:**
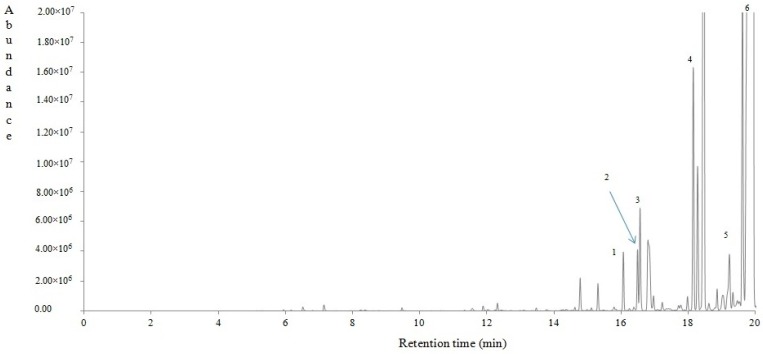

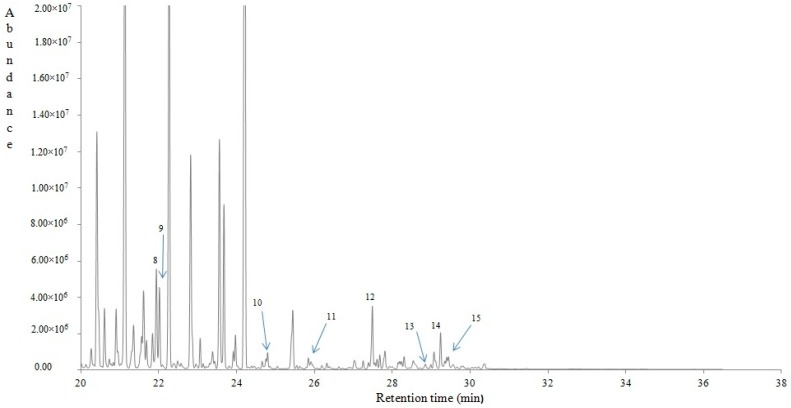
A typical gas chromatogram of *Lavandula stoechas*. Indicative volatile compounds (markers) are numbered according to the retention time shown in Table 2. 1: alpha-Pinene, 2: alpha-Fenchene, 3: Camphene, 4: para-Cymene, 5: cis-Linalool oxide, 6: alpha-Thujone, 7: Camphor, 8: alpha-Terpineol, 9: Myrtenal, 10: cis-Geranyl acetate, 11: (+)-Sativene, 12: beta-Selinene, 13: Viridiflorol, 14: (+)-Aromadendrene.

Fifty volatile compounds were tentatively identified using HS-SPME/GC-MS, belonging to alcohols, aldehydes, ketones, norisoprenoids and numerous terpenoids. In Table 2 is given (among other data) the percentage contribution of each volatile compound to the total ones. Figure 3 shows a typical gas chromatogram of *Lavandula stoechas*, pointing with numbers to some indicative volatile compounds. The most abundant volatiles were a-thujone (32.14%), 1,3,3-trimethyl-2-oxabicyclo[2.2.2] octane (9.77%), (+)-myrtenyl acetate (7.75%), camphor (6.07%), 1-octen-3-yl-acetate (3.94%) and fenchyl acetate (3.91%), followed by minor proportions of numerous others. A considerable number of these compounds have been reported in the essential oil of plants with edible modified stems, roots and bulbs in the Solanaceae, Tropacolaceae, Typhaceae and Zingiberaceae families, which are grown all over the world [42].

The biosynthesis of thujone in plants starts with the formation of geranyl diphosphate (GPP) from dimethylallyl pyrophosphate (DMAPP) and isopentenyl diphosphate (IPP), catalyzed by the enzyme geranyl diphosphate synthase [43]. In addition, Umlauf and Zapp [44], using carbon nuclear magnetic spectroscopy (^13^C-NMR), reported that the isoprene units used to form thujone in plants are derived from an alternative pathway, the methylerythritol phosphate pathway (MEP).

Different opinions occur regarding the use of thujone as a food or drink supplement. In particular, the maximum thujone levels in the E.U. are related to the Artemisia species used along with the matrix (food or beverage). In that sense, the respected range may be within 0.5–35 mg kg^−1^ [45,46].

On the other hand, in the U.S., the addition of pure thujone to foods is not permitted [47]. Foods or beverages that contain Artemisia species, white cedar, oak moss, tansy or yarrow must be free of thujone [48], which practically means that these contain less than 10 mg L^−1^ of thujone [49]. Sage and sage oil (which may contain up to 50% thujone) are on the Food and Drug Administration’s list of generally recognized as safe (GRAS) substances [50]. The average lethal dose value, or LD_50_, of alpha-thujone, the more active of the two isomers (alpha- and beta-) found in plant-derived products, has been reported to be ca. 45 mg kg^−1^ in mice, with a 0% mortality rate at 30 mg kg^−1^ and a 100% mortality rate at 60 mg kg^−1^, respectively [51].

The biochemical pathway of camphor production involves the presence of geranyl pyrophosphate, which, via the cyclisation of linaloyl pyrophosphate, turns to bornyl pyrophosphate, followed by hydrolysis to borneol and then oxidation to camphor. Camphor has been used in traditional medicine since ancient times in countries where it was native. Its characteristic odour and its decongestant effect on the treatment of sprains, swellings and inflammation have probably led to its use in medicine [52,53]. Camphor was also used for centuries in Chinese medicine for a variety of health issues. The lethal doses in adults are in the range of 50–500 mg kg^−1^ after oral exposure. In particular, 2 g may cause serious toxicity and 4 g is potentially lethal [54]. To the best of our knowledge, this is the first report in the literature reporting the volatile profile of Greek *Lavandula stoechas*.

## 5. Conclusions

*Lavandula stoechas* from the region of Saidona proved to be a good source of phytochemicals with a high in vitro antioxidant activity, especially in its aqueous form. In addition, numerous terpenoids were tentatively identified, producing a strong, penetrating and menthol-like odour. The mode of mechanistic action by the plant may involve its cultivation and exploitation under the prism of natural ‘’medicinal agents’’ that may favor the healthcare of humans. In parallel, the significance of its use as a supplement by the food industry or related industries in specific amounts may favor the preparation and distribution of health-promoting foods and beverages (HPFB) of a distinct flavour. Future research will be focused on the determination of the cytotoxicity of *Lavandula stoechas* dried proportions or aqueous extracts used in the present work (ca. 1%, *w v*^−1^), based on in vivo measurements, to approve or not its prospective use in different forms in foods or beverages.

## Figures and Tables

**Table 1:** Phytochemicals identified in methanolic and aqueous extracts of Ladastacho.

Peak Number	RT (min)	[M−H]^−^ (*m*/*z*)	Phytochemicals
1	3.0	375(100), 573(46), 751(38)	5-nonadecylresorcinol
2	4.0	387(100), 607(30)	Unknown
3	8.6	179(100), 135(25)	Caffeic acid
4	11.1	473(100)	6′′-*O*-Acetylgenistin
5	15.8	463(100), 485(20)	Quercetin 3-*O*-glucoside
6	16.1	461(100), 285(3)	Luteolin-*O*-glucuronide
7	16.6	447(100)	Luteolin-*O*-glucoside
8	17.7	359(100), 719(86)	Rosmarinic acid isomer
9	19.2	445(100), 269(5)	Apigenin-*O*-glucuronide
10	19.4	359(100), 719(30)	Rosmarinic acid
11	20.7	549(100), 507(94)	Salvianolic acid derivative
12	22.1	717(100), 519(43)	Salvianolic acid B (Lithospermic acid B)

RT, retention time (minutes).

**Table 2:** Volatile compounds identified in *Lavandula stoechas*

RT (min)	KI	RT_lit_	Contribution (%)	Volatile compounds	Qualification	MOI
6.51	<800	<800	0.06	3-Buten-2-one (Vinyl methyl ketone)	91	MS/KI
7.14	<800	<800	0.08	3-Buten-2-ol, 2-methyl	90	MS/KI
12.31	<800	<800	0.08	Hexanal	95	MS/KI
14.62	885	891	0.04	2-Heptanone	94	MS/KI
15.31	913	915	0.28	3,3-Dimethylallyl acetate	81	MS/KI
15.78	932	-	0.04	2,4,4-Trimethylcyclopentanone	94	MS/KI
16.06	944	937	0.44	(1S)-2,6,6-Trimethylbicyclo[3.1.1]hept-2-ene (apha-Pinene)	96	MS/KI
16.48	962	958	0.48	(−)-7,7-Dimethyl-2-methylenebicyclo[2.2.1]heptane (alpha-Fenchene)	97	MS/KI
16.56	965	964	0.89	2,2-Dimethyl-3-methylidenebicyclo[2.2.1]heptane (Camphene)	97	MS/KI
16.96	982	985	0.16	3-Octanone	96	MS/KI
17.22	993	980	0.07	6,6-dimethyl-2-methylene-Bicyclo[3.1.1]heptane	95	MS/KI
18.14	1034	1023	2.36	1-methyl-4-(1-methylethyl)-Benzene	97	MS/KI
18.27	1039	1035	1.19	1-methyl-4-(prop-1-en-2-yl)Cyclohex-1-ene (dl-Limonene)	99	MS/KI
18.45	1048	1044	9.77	1,3,3-trimethyl-2-oxabicyclo[2.2.2]Octane	98	MS/KI
18.85	1066	1068	0.19	1-methyl-4-(1-methylethyl)-1,4-Cyclohexadiene	97	MS/KI
19.20	1082	1080	1.05	2-Furanmethanol, 5-ethenyltetrahydro-.alpha.,.alpha.,5-trimethyl-, cis- (cis-Linalool oxide)	91	MS/KI
19.31	1086	1096	0.19	[(1S,4S)-4-methyl-1-propan-2-yl-4-bicyclo[3.1.0]hexanyl] acetate (trans-Sabinene hydrate)	96	MS/KI
19.44	1092	-	0.28	3,4,4-trimethyl-2-Cyclohexen-1-one	83	MS/KI
19.51	1096	1099	0.10	1-methyl-4-(1-methylethylidene)-Cyclohexene	98	MS/KI
19.59	1099	1110	3.94	1-Octen-3-yl acetate	90	MS/KI
19.86	1112	1117	32.14	α: (1S,4R,5R)-4-methyl-1-(propan-2-yl)bicyclo[3.1.0]Hexan-3-one (alpha-Thujone)	95	MS/KI
20.40	1139	1121	2.80	(1R,3S,4S)-2,2,4-trimethylbicyclo[2.2.1]heptan-3-ol (alpha- Fenchyl alcohol)	97	MS/KI
21.12	1174	1146	6.07	1,7,7-Trimethylbicyclo[2.2.1]Heptan-2-one (Camphor)	97	MS/KI
21.34	1185	1164	0.66	6,6-dimethyl-4-methylidenebicyclo[3.1.1]Heptan-3-one (Pinocarvone)	81	MS/KI
21.55	1195	1164	0.40	endo-1,7,7-Trimethyl- bicyclo[2.2.1]Heptan-2-ol (Borneol)	93	MS/KI
21.67	1201	1183	0.27	1-(3-methylphenyl)-Ethanone	95	MS/KI
21.83	1209	1195	0.35	.alpha.,.alpha.4-trimethyl-3-Cyclohexene-1-methanol (alpha-Terpineol)	91	MS/KI
22.01	1219	1195	0.73	6,6-Dimethylbicyclo[3.1.1]hept-2-ene-2-carboxaldehyde (Myrtenal)	97	MS/KI
22.26	1232	1223	3.91	(2,2,4-trimethyl-3-bicyclo[2.2.1]Heptanyl) acetate (Fenchyl acetate)	96	MS/KI
23.26	1284	1280	0.11	4-Isopropenyl-1-methyl-7-oxabicyclo[4.1.0]heptan-2-one (trans-Carvone oxide)	88	MS/KI
23.55	1300	1285	2.04	Bicyclo[2.2.1]heptan-2-ol, 1,7,7-trimethyl-, acetate, (1S-endo)-	98	MS/KI
23.67	1306	-	1.61	1-methyl-4-(1-methylethylidene)-Cyclohexanol (γ-Terpineol)	91	MS/KI
23.91	1320	-	0.15	1-methylene-4-(1-methylethenyl)-Cyclohexane (Pseudolimonene)	85	MS/KI
23.96	1322	-	0.32	2,2-dimethyl-3-methylene- Bicyclo[2.2.1]heptane [Camphene, (1R,4S)-(+)-]	91	MS/KI
24.20	1336	1332	7.75	(6,6-Dimethylbicyclo[3.1.1]hept-2-en-2-yl)methyl acetate [(-)-Myrtenyl acetate]	87	MS/KI
24.75	1367	1352	0.06	1H-Cyclopenta[1,3]cyclopropa[1,2]benzene, 3a,3b,4,5,6,7-hexahydro-3,7-dimethyl-4-(1-methylethyl)-, [3aS-(3aα,3bβ,4β,7α,7aS*)-(-)-] (alpha-Cubebene)	99	MS/KI
24.79	1369	1369	0.09	2,6-Octadien-1-ol, 3,7-dimethyl-, acetate, (Z)- [cis-Geranyl acetate)	91	MS/KI
25.44	1405	1378	0.61	1,2,4-Metheno-1H-indene, octahydro-1,7a-dimethyl-5-(1-methylethyl)-, [1S-(1.alpha.,2.alpha.,3a.beta.,4.alpha.,5.alpha.,7a.beta.,8S*)]-[(+)-Cyclosativene]	94	MS/KI
25.84	1429	1396	0.07	1,4-Methano-1H-indene, octahydro-4-methyl-8-methylene-7-(1-methylethyl)-, [1S-(1.alpha.,3a.beta.,4.alpha.,7.alpha.,7a.beta.)]- [(+)-Sativene]	99	MS/KI
27.25	1515		0.08	2,3-dihydro-1,3,3-trimethyl-2-methylene-1H-Indole	80	MS/KI
27.39	1523	1505	0.06	Naphthalene, 1,2,4a,5,6,8a-hexahydro-4,7-dimethyl-1-(1-methylethyl)-, (1S,4aS,8aR)- (alpha-Muurolene)	98	MS/KI
27.49	1530	1509	0.62	Naphthalene, decahydro-4a-methyl-1-methylene-7-(1-methylethenyl)-, [4aR-(4a.alpha.,7.alpha.,8a.beta.)]-(beta-Selinene)	99	MS/KI
27.55	1534	1505	0.05	alpha-Eudesma-3,11-diene (alpha-Selinene)	99	MS/KI
27.68	1542	1540	0.12	Naphthalene, 1,2,3,5,6,8a-hexahydro-4,7-dimethyl-1-(1-methylethyl)-, (1S-cis)-	99	MS/KI
28.30	1581	1525	0.13	Naphthalene, 1,2,3,4,4a,7-hexahydro-1,6-dimethyl-4-(1-methylethyl)-	87	MS/KI
28.85	1617	-	0.08	4aH-Cycloprop[e]azulen-4a-ol, decahydro-1,1,4,7-tetramethyl-, [1aR-(1a.alpha.,4.beta.,4a.beta.,7.alpha.,7a.beta.,7b.alpha.)]- (Palustrol)	96	MS/KI
29.07	1631	1613	0.21	(4,6)]dodecane,,12-trimethyl-9-methylene-, [1R-(1R*,4R*,6R*,10S*)]- (CAS); (-)-.beta.-Caryophyllene epoxide	91	MS/KI
29.24	1643	1620	0.39	1H-Cycloprop[e]azulen-4-ol, decahydro-1,1,4,7-tetramethyl-, [1ar-(1a.alpha.,4.beta.,4a.beta.,7.alpha.,7a.beta.,7b.alpha.)]- (Viridiflorol)	99	MS/KI
29.40	1653	-	0.11	1H-Cycloprop[e]azulene, decahydro-1,1,7-trimethyl-4-methylene-, [1aR-(1a.alpha.,4a.alpha.,7.alpha.,7a.beta.,7b.alpha.)]- [(+)-Aromadendrene]	94	MS/KI

RT: retention time (minutes), KI: experimental retention indices values based on the calculations of Kovats index values (KI) using the standard mixture of alkanes. RI_lit_: Retention indices of the identified compounds according to literature data included in Wiley 7 NIST MS library. Qualification: Percentage accuracy of volatile compounds identified using Wiley 7 NIST MS library data. Results reported are the average ± standard deviations values of two independent replicates (*n* = 2).
